# Thyroid V40 as a potential early predictor of hypothyroidism following hypofractionated locoregional breast radiotherapy

**DOI:** 10.1371/journal.pone.0351856

**Published:** 2026-06-18

**Authors:** Moemen Khalifa, Maher Soliman, Abbas Omar, Mohamed Abouegylah, Amr Munir Ameen, Yomna Mahmoud Mohamad, Waleed Arafat

**Affiliations:** 1 Department of Clinical Oncology and Nuclear Medicine, Faculty of Medicine, Alexandria University, Alexandria, Egypt; 2 Alexandria Comprehensive Cancer Center, Alexandria, Egypt; All India Institute of Medical Sciences, INDIA

## Abstract

Radiotherapy (RT) is used quite often among breast cancer patients, and because these patients now live longer than they used to, long-term treatment-related adverse effects, such as hypothyroidism, are becoming increasingly relevant. Radiation-induced hypothyroidism is a late side effect of radiation to the thyroid gland, which could develop months to years after radiotherapy. However, there are insufficient data on hypothyroidism in patients with breast cancer who receive locoregional RT, which usually affects a portion of the thyroid gland. In this prospective study, we aimed to evaluate the association between radiation dose to the thyroid gland and early thyroid dysfunction in breast cancer patients treated with hypofractionated locoregional radiotherapy, and to establish potential planning objectives that could help in sparing the thyroid gland. Our study included 109 women who received 3-D conformal locoregional breast radiotherapy. These patients had normal thyroid function prior to start of radiotherapy. Patients had follow-up thyroid function tests 6 months after finishing radiotherapy. Based on dose volume histograms (DVH), the percentages of the thyroid volume, and ipsilateral thyroid lobe, receiving 5, 10, 15, 20, 25, 30, 35, and 40 Gy (V5, V10, V15, V20, V25, V30, V35, and V40 respectively), in addition to the mean and maximum thyroid doses, were estimated. Follow-up assessment of thyroid function and statistical analysis unveiled incidence of radiotherapy induced hypothyroidism to be 8.3%. Possible risk factors included high maximum radiation dose and V40 to the thyroid gland and the ipsilateral thyroid lobe, as well as high mean dose and smaller volume of the ipsilateral thyroid lobe. The likelihood of hypothyroidism was significantly increased with V40 of >5% of the thyroid gland, and V40 of >11% of the ipsilateral thyroid lobe. Further study is recommended for larger populations and longer follow-up periods.

## Introduction

External beam radiotherapy plays an important role in the management of breast cancer, especially after surgery and chemotherapy. There are different types of breast radiotherapy, like intraoperative radiotherapy (IORT), 3D-conformal radiotherapy (3D-CRT), intensity-modulated radiotherapy (IMRT), and brachytherapy. While 3D-CRT has been the standard, IMRT is gaining attention for its ability to target the tumor area more precisely, especially in left-sided breast cancer, where it can reduce radiation exposure to the heart [1,2]. In recent years, there's been a shift towards a shorter radiation treatment schedule called hypofractionation. This approach delivers higher doses of radiation in a shorter time, typically 40.05 Gy over three weeks, with or without a boost. Hypofractionation has become the standard for early-stage breast cancer because it offers similar results to traditional methods but with fewer side effects [3]. Radiotherapy to the supraclavicular lymph nodes is usually indicated in patients found to have deposits of malignant cells in the regional lymph nodes [4,5].

Hypothyroidism is classified into clinical and subclinical hypothyroidism, according to laboratory results and the presence of symptoms. Clinical hypothyroidism is characterized by an increase in the thyroid stimulating hormone (TSH) with an associated reduction in thyroxine (T4) and the presence of clinical symptoms, such as weight gain, fatigue, impaired wound healing, cold intolerance and so on. On the other hand, subclinical hypothyroidism is generally defined as an increase in TSH paired with a normal level of circulating T4 and the absence of symptoms noticed by the patient [6].

Thyroid gland injury by ionizing radiation is due to a variety of pathological mechanisms. These mechanisms include vascular effects in the epithelium of small vessels and fibrosis of capsular structures, while the damage to the follicular epithelial cells is considered of lesser importance [3,7]. Ultrasonography has proven that changes in both the blood vessels and the gland echogenicity occur during radiotherapy, and the subsequent development of acute thyroiditis was correlated to vessel changes [8]. The most commonly found late morphological changes consist of atrophy, chronic inflammation (thyroiditis) with lymphocytic infiltration, vascular fibrosis, and focal and irregular follicular hyperplasia [9,10]. The mechanisms for radiation-induced hypothyroidism are uncertain, however they are thought to be linked to vascular damage [7]. Radiation induced hypothyroidism usually develops at a median of 9 months after treatment (ranging from 3 months to 7.2 years), and following doses equal to or higher than 40 gray [11–16].

The growing longevity of breast cancer patients has brought attention to the importance of understanding treatment-related late adverse effects. In the context of head and neck cancer and Hodgkin lymphomas, the development of primary hypothyroidism is a recognized late adverse effect following curative radiation therapy (RT) targeting the neck region.[7,17–19] Notably, the radiation fields used in these cases encompass the entire thyroid gland.[18,20–24] However, in breast radiotherapy, only a portion of the thyroid gland is exposed to radiation. Yet this side effect has not been extensively studied within the realm of breast irradiation.

## Methods and materials

This prospective study included 109 women with non-metastatic breast cancer, who received post-operative loco-regional breast radiotherapy at the Alexandria University main hospital. Our inclusion criteria were female patients older than 18 years of age, patients with normal thyroid functions prior to radiotherapy, patients with no history of hypothyroidism, hyperthyroidism, or thyroidectomy, patients who were unavailable for follow-up or declined testing were excluded.

This prospective observational study was approved by the Ethics Committee of Alexandria University (IRB NO: 00012098). Informed consent was obtained from all participants and was documented in patients’ medical records by the treating physician.. Recruitment started on 15^th^ of January 2022 and ended on 1^st^ of May 2022.

Baseline thyroid function tests: thyroid stimulating hormone (TSH), free thyroxine (FT4), and free triiodothyronine (FT3).

TSH; normal range; 0.35–5 µU/mL), (FT4; normal range, 0.89–1.76 ng/dL), and triiodothyronine (T3; normal range, 2.3–4.2 pg/dl).

Follow up thyroid function tests were repeated 6 months after finishing RT.

Hypothyroidism was defined according to the American Thyroid Association recommendations [25] as TSH level more than the normal laboratory range, regardless of symptoms.

### Simulation

The patients were simulated supine on a breast board with arms bilaterally abducted and externally rotated and head slightly tilted to the opposite side of the affected breast [26].

Computed tomography scans were obtained at 5-mm slice intervals and sent to the planning system for contouring.

### Contouring

Clinical target volume (CTV) and ipsilateral supraclavicular lymph node were delineated according to RTOG guidelines. The ipsilateral lung, the heart and the spinal cord were contoured as organs at risk (OAR) as per RTOG OAR atlas as well [27].

The thyroid gland, as well as the ipsilateral thyroid lobe, were contoured manually on the computed tomography slices.

### Planning

All patients were treated using 3-D conformal hypofractionated loco-regional radiotherapy (40.05 Gy in 15 fractions).

Treatment was performed with photons using a 6- and 15-MV linear accelerator.

Patients were treated using a mono-isocenter, with medial and lateral tangential fields for the breast/chest wall, and either an anterior, or a combination of an anterior and a posterior field for the supraclavicular lymph node field, with a slight angle of 10–15 degrees. Both 6 and 15 MV energies were used according to separation and breast size of patients.

Plans were optimized so that the target receives 95–105% of the dose, while keeping OAR within their acceptable tolerance levels [28].

Dose volume histograms were created for all treatment plans and all dosimetric data were transferred to 3-D radiotherapy planning system.

The absolute thyroid volume, doses to thyroid gland (mean, maximum and minimum) and the percentage of thyroid volume receiving 10, 15, 20, 25, 30, 35, 40 Gy (V10, V15, V20, V25, V30, V35, V40) were analyzed from Dose volume histograms (DVH).

### Interpretation

Patients were grouped into euthyroid and hypothyroid groups, with patients showing TSH levels of > 5 µU/mL being considered hypothyroid, then descriptive analyses were done for comparison between the groups, and further multivariate regression analysis was carried out to pin point the most significant elements. Receiver operating characteristic (ROC) curves were used to identify clinically significant cut-off points. All analyses had p-values <0.05 indicating significance.

Due to the limited number of events (n = 9), multivariate regression analysis may be underpowered and prone to overfitting. Therefore, results from multivariate analysis should be interpreted with caution, and greater emphasis was placed on univariate and ROC analyses.

## Results

Our patients had a median age of 54, ranging from 24 to 81 years old. Left and right sided tumors were almost equal, with right side representing 47.7% of the cases and left side representing the remaining 52.3%. Table 1.

**Table 1:** Patients and treatment characteristics.

	Total (n = 109)	
	No.	%
**Age (years)** Median (IQR)	54.0 (45.0 – 60.0)	
**Side**		
Right	52	47.7
Left	57	52.3
**Type of surgery**		
SSM	6	5.5
MRM	66	60.6
BCS	37	33.9
**Chemotherapy**		
Adjuvant	68	62.3
Neoadjuvant	38	34.9
None	3	2.8
**Hormonal therapy**		
Yes	81	74.3
No	28	25.7
**HR**		
Negative	28	25.7
Positive	81	74.3
**HER2**		
Negative	73	67.0
Positive	36	33.0
**Stage**		
IIA	11	10.1
IIB	48	44.0
IIIA	33	30.3
IIIB	4	3.7
IIIC	13	11.9

SSM: Skin sparing mastectomy MRM: Modified radical mastectomy BCS: Breast conserving surgery HR: Hormone receptors HER2: Human epidermal growth factor receptor 2

At the end of study, 9 patients (8.3%) developed hypothyroidism.

Different volumes of the thyroid gland were compared to see if smaller or larger thyroid glands were more prone to developing radiation induced hypothyroidism. However, there was no statistically significant difference shown between the size of the thyroid gland and the incidence of hypothyroidism.

**Fig 1:**
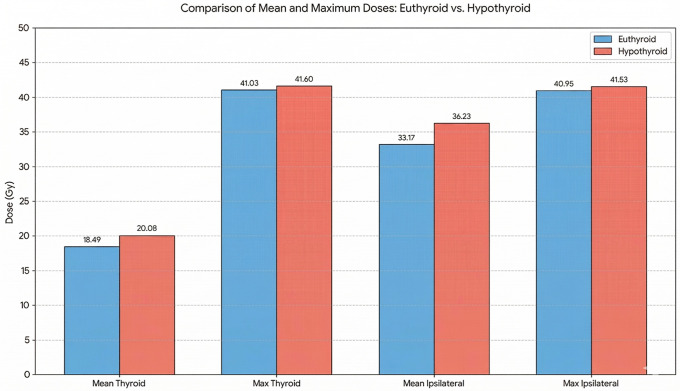
Comparison of mean and maximum radiation doses (Gy) delivered to the thyroid gland and ipsilateral lobe in euthyroid versus hypothyroid groups.

Different dose-volumetric parameters were compared to determine which parameters correlated with the incidence of hypothyroidism. The volume that received 40 Gy in hypothyroid patients was 10.36% compared to 5.67% in euthyroid patients and the difference was significant (p value = 0.030). Fig 1

The volume of the thyroid receiving 40 Gy in cc showed a statistically significant difference, its mean being 0.48 cc in the euthyroid group and 1.08 cc in the hypothyroid group with a p value of 0.013. Table 2

**Table 2:** Dosimetric values for thyroid gland.

Thyroid	Total (n = 109)	TSH		Test of Sig.	p
		Euthyroid (n = 100)	Hypothyroid (n = 9)		
**Mean dose (Gy)** Mean ± SD.	18.62 ± 4.36	18.49 ± 4.41	20.08 ± 3.73	t = 1.049	0.297
**Maximum dose (Gy)** Mean ± SD.	41.07 ± 0.80	41.03 ± 0.79	41.60 ± 0.63	t= 2.085^*^	0.039^*^
**Minimum dose (Gy)** Mean ± SD.	1.08 ± 0.43	1.09 ± 0.44	1.04 ± 0.28	U = 448.0	0.982
**V5 (%)** Mean ± SD	55.04 ± 9.28	54.71 ± 9.38	58.68 ± 7.66	t = 1.232	0.221
**V10 (%)** Mean ± SD.	51.30 ± 9.29	51.05 ± 9.35	54.03 ± 8.59	t = 0.921	0.359
**V15 (%)** Mean ± SD.	49.18 ± 9.42	48.91 ± 9.43	52.20 ± 9.20	t = 1.003	0.318
**V20 (%)** Mean ± SD.	46.86 ± 9.53	46.54 ± 9.48	50.41 ± 9.88	t = 1.168	0.245
**V25 (%)** Mean ± SD.	43.67 ± 10.0	43.28 ± 9.89	48.03 ± 10.83	t = 1.373	0.173
**V30 (%)** Mean ± SD.	38.67 ± 10.95	38.17 ± 10.73	44.17 ± 12.46	t = 1.586	0.116
**V35 (%)** Mean ± SD.	28.83 ± 11.56	28.23 ± 11.21	35.46 ± 13.89	t = 1.817	0.072
**V40 (%)** Mean ± SD.	6.06 ± 7.43	5.67 ± 7.33	10.36 ± 7.53	U = 252.50^*^	0.030^*^
**V5 (cc)** Mean ± SD.	4.78 ± 2.76	4.60 ± 2.59	6.77 ± 3.88	U = 301.0	0.101
**V10 (cc)** Mean ± SD.	4.48 ± 2.64	4.32 ± 2.48	6.23 ± 3.71	U = 318.0	0.146
**V15 (cc)** Mean ± SD.	4.32 ± 2.62	4.17 ± 2.46	6.04 ± 3.70	U = 317.0	0.143
**V20 (cc)** Mean ± SD.	4.15 ± 2.57	3.99 ± 2.42	5.85 ± 3.67	U = 312.0	0.129
**V25 (cc)** Mean ± SD.	3.89 ± 2.50	3.74 ± 2.34	5.56 ± 3.59	U = 310.0	0.123
**V30 (cc)** Mean ± SD.	3.47 ± 2.34	3.32 ± 2.18	5.09 ± 3.46	U = 302.0	0.103
**V35 (cc)** Mean ± SD.	2.59 ± 1.93	2.46 ± 1.77	4.03 ± 3.01	U = 291.0	0.080
**V40 (cc)** Mean ± SD.	0.53 ± 0.73	0.48 ± 0.70	1.08 ± 0.95	U = 224.0^*^	0.013^*^

The same dose-volumetric parameters were used again, but this time with only the ipsilateral thyroid lobe, instead of the whole thyroid gland.

Ipsilateral thyroid lobes with a smaller size strongly correlated with higher incidence of hypothyroidism (p value = 0.034). Table 3.

**Table 3:** Comparison between Euthyroid and Hypothyroid according to ipsilateral thyroid lobe volume.

Ipsilateral thyroid lobe	Total (n = 109)		TSH				Test of Sig.	p
			Euthyroid (n = 100)		Hypothyroid (n = 9)			
	No.	%	No.	%	No.	%		
**Volume in cc**								
<5	45	41.3	39	39.0	6	66.7	χ^2^= 5.988^*^	^MC^p = 0.034^*^
5–10	52	47.7	51	51.0	1	11.1		
>10	12	11.0	10	10.0	2	22.2		
Range	1.22–32.87		1.22–32.87		1.97–27.66			
Mean ± SD.	6.55 ± 4.71		6.53 ± 4.30		6.76 ± 8.34		U = 291.0	0.080
Median (IQR)	5.48 (3.81–7.74)		5.74 (3.92–7.82)		2.77 (2.32–5.64)			

The mean dose to the ipsilateral thyroid lobe also demonstrated a statistically significant difference, with a mean of 33.17 Gy and 36.23 Gy in the euthyroid and hypothyroid groups respectively (p value = 0.043) Table 4.

**Table 4:** Dosimetric analysis for ipsilateral thyroid lobe.

Thyroid	Total (n = 109)	TSH		Test of Sig.	p
		Euthyroid (n = 100)	Hypothyroid (n = 9)		
**Mean dose (Gy)** Mean ± SD.	33.43 ± 4.36	33.17 ± 4.42	36.23 ± 2.35	t = 2.046^*^	0.043^*^
**Maximum dose (Gy)** Mean ± SD.	41.0 ± 0.81	40.95 ± 0.81	41.53 ± 0.69	t = 2.097^*^	0.038^*^
**Minimum dose (Gy)** Mean ± SD.	11.10 ± 9.26	10.67 ± 9.25	15.94 ± 8.30	U = 285.0	0.069
**V5 (%)** Mean ± SD.	97.63 ± 5.35	97.43 ± 5.55	99.90 ± 0.30	U = 300.0	0.059
**V10 (%)** Mean ± SD.	96.0 ± 7.58	95.66 ± 7.83	99.75 ± 0.60	U = 281.0^*^	0.046^*^
**V15 (%)** Mean ± SD.	94.26 ± 9.27	93.79 ± 9.54	99.40 ± 0.98	U = 269.50^*^	0.044^*^
**V20 (%)** Mean ± SD.	91.67 ± 11.15	91.07 ± 11.44	98.42 ± 1.58	U = 259.0^*^	0.035^*^
**V25 (%)** Mean ± SD.	87.20 ± 13.89	86.44 ± 14.20	95.69 ± 4.77	t = 4.339^*^	<0.001^*^
**V30 (%)** Mean ± SD.	78.73 ± 18.09	77.80 ± 18.33	89.03 ± 11.48	U = 276.0	0.055
**V35 (%)** Mean ± SD.	59.73 ± 22.11	58.61 ± 22.06	72.14 ± 19.76	t = 1.776	0.079
**V40 (%)** Mean ± SD.	12.31 ± 14.84	11.55 ± 14.69	20.83 ± 14.63	U = 259.50^*^	0.036^*^
**V5 (cc)** Mean ± SD.	20.66 ± 11.97	19.84 ± 11.14	29.71 ± 17.26	U = 288.0	0.074
**V10 (cc)** Mean ± SD.	20.43 ± 12.09	19.60 ± 11.26	29.70 ± 17.27	U = 288.0	0.074
**V15 (cc)** Mean ± SD.	20.18 ± 12.18	19.33 ± 11.35	29.63 ± 17.27	U = 286.0	0.071
**V20 (cc)** Mean ± SD.	19.76 ± 12.22	18.89 ± 11.41	29.38 ± 17.13	U = 283.0	0.066
**V25 (cc)** Mean ± SD.	18.94 ± 12.03	18.07 ± 11.25	28.57 ± 16.59	U = 275.0	0.054
**V30 (cc)** Mean ± SD.	17.24 ± 11.45	16.41 ± 10.72	26.43 ± 15.59	U = 275.0	0.054
**V35 (cc)** Mean ± SD.	13.11 ± 9.66	12.37 ± 8.83	21.32 ± 14.51	U = 292.0	0.082
**V40 (cc)** Mean ± SD.	2.63 ± 3.75	2.32 ± 3.21	6.16 ± 6.85	U = 254.0^*^	0.031^*^

**Fig 2:**
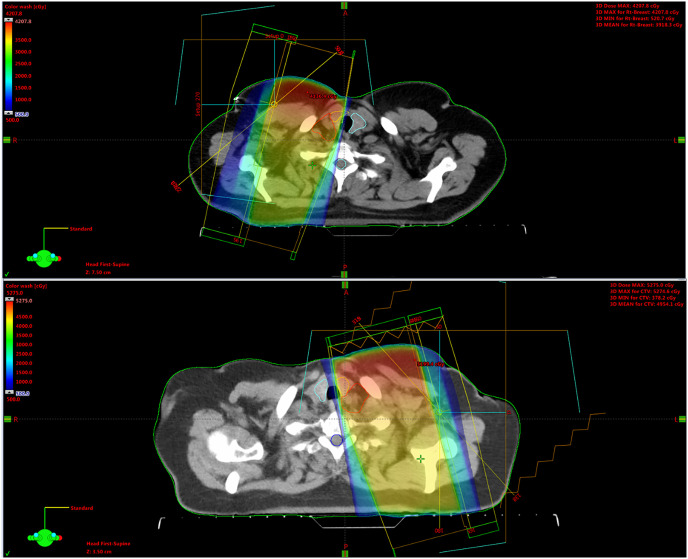
Color-wash dose distribution across the ipsilateral lobe.

Additionally, the maximum dose received by the ipsilateral thyroid lobe was also statistically significant, being 40.95 Gy in the euthyroid group compared to 41.53 Gy in the hypothyroid group. Fig 2

The V10, V15, V20, V25, and V40 all showed a significant difference between the two groups, indicating that the incidence of hypothyroidism indeed correlates with higher doses received by the ipsilateral thyroid lobe (p value of 0.046, 0.044, 0.035, < 0.001, and 0.036 respectively). While only the V40 in cc showed a significant difference when comparing the two groups, with a mean of 2.32 cc in the euthyroid group and 6.16 cc in the hypothyroid group, with a p value of 0.031.

### Multivariate analysis and interpretation

Multivariate analysis between statistically significant parameters showed that the most impactful were the percentage volume of the thyroid and ipsilateral thyroid lobe receiving 40 Gy, as well as the ipsilateral thyroid lobe volume in cc receiving 40 Gy (p values of 0.046, 0.044 and 0.031 respectively). Table 5

**Table 5:** Multivariate Logistic regression analysis for dose-volumetric parameters affecting Hypothyroid.

	p	OR (LL – UL 95%C.I)
**Thyroid max. (Gy)**	0.898	0.579 (0.000–2517.4)
**Thyroid V40 (%)**	0.046^*^	2.432 (1.015–5.826)
**Thyroid V40 (cc)**	0.067	0.042 (0.001–1.242)
**Ipsilateral thyroid mean (Gy)**	0.622	0.638 (0.106–3.826)
**Ipsilateral thyroid Max. (Gy)**	0.618	8.255 (0.002–33415)
**Ipsilateral thyroid V10 (%)**	0.680	2.130 (0.059–77.541)
**Ipsilateral thyroid V15 (%)**	0.972	0.944 (0.039–22.826)
**Ipsilateral thyroid V20 (%)**	0.864	1.207 (0.139–10.503)
**Ipsilateral thyroid V25 (%)**	0.889	1.071 (0.410–2.796)
**Ipsilateral thyroid V40 (%)**	0.044^*^	0.617 (0.386–0.986)
**Ipsilateral thyroid V40 (cc)**	0.031^*^	2.266 (1.078–4.764)

**Fig 3:**
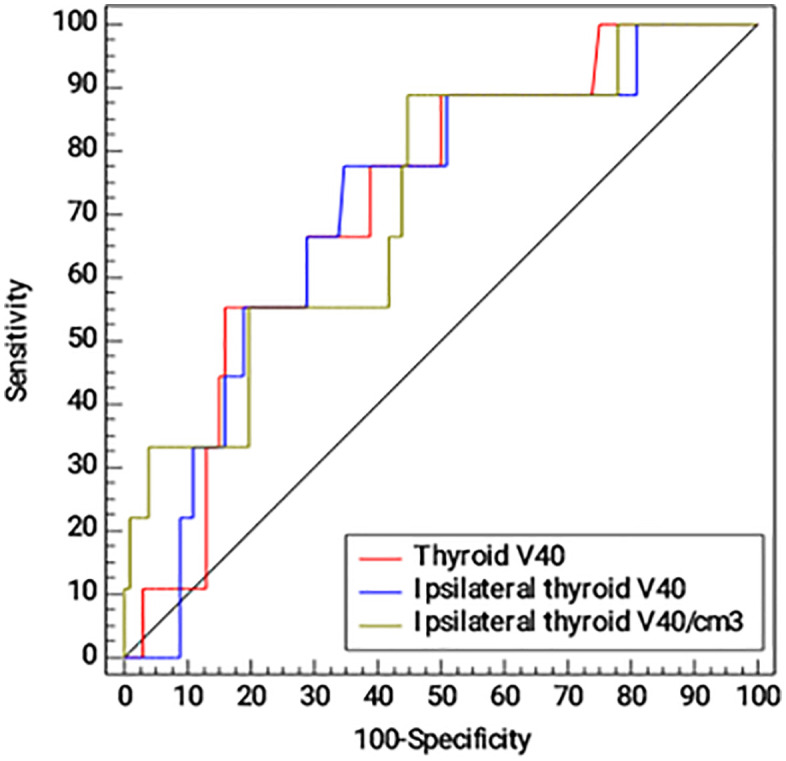
ROC curve for Thyroid V40 (%), Ipsilateral thyroid V40 (%), and Ipsilateral thyroid V40 (cc) to predict Hypothyroid patients (n = 9) from Euthyroid (n = 100).

Receiver operating characteristic (ROC) curves were used to identify cut-off points for these values, showing significance when thyroid V40 was higher than 5% with p value of 0.03, and ipsilateral thyroid lobe V40 more than 11% with p value of 0.036. Fig 3

As well as when more than 1 cc of the ipsilateral thyroid lobe received 40 Gy (p value = 0.031). Predictive performance was modest (AUC 0.71), with low PPV (15–17%) and higher NPV. Table 6

**Table 6:** Prognostic performance for Thyroid V40 (%), Ipsilateral thyroid V40 (%), and Ipsilateral thyroid V40 (cc) to predict Hypothyroid patients (n = 9) from Euthyroid (n = 100).

	AUC	p	95% C.I	Cut off	Sensitivity	Specificity	PPV	NPV
**Thyroid V40 (%)**	0.719	0.030^*^	0.567–0.872	>5	77.78	61.0	15.2	96.8
**Ipsilateral thyroid V40 (%)**	0.712	0.036^*^	0.552–0.871	>11^#^	77.78	65.0	16.7	97.0
**Ipsilateral thyroid V40 (cc)**	0.718	0.031^*^	0.548–0.887	>1^#^	88.89	55.0	15.1	98.2

AUC: Area Under a Curve; p value: Probability value; *: Statistically significant at p ≤ 0.05; CI: Confidence Intervals; #Cut off was chosen according to Youden index; NPV: Negative predictive value; PPV: Positive predictive value

## Discussion

The National Comprehensive Cancer Network (NCCN) recommends that thyroid function tests should be repeated every 6–12 months after radiotherapy (RT) for neck, as radiation induced hypothyroidism is a common complication following radiotherapy in head and neck cancers. However, in breast cancer loco-regional radiotherapy, the thyroid hormone level before and after RT are not usually assessed in routine clinical practice as there is no clear consensus established yet [29].

The main aim of this study was to assess the incidence of early thyroid dysfunction at 6 months in breast cancer patients treated with loco-regional radiotherapy as the supraclavicular radiation field usually included a big portion of the thyroid gland. And to investigate predictors affecting the development of hypothyroidism after radiotherapy for breast cancer patients, focusing on radiation dose-volumetric parameters. Therefore, we analyzed the information of 109 patients who received loco-regional breast radiotherapy using 3D-conformal technique at the Clinical Oncology and Nuclear Medicine Department (ACOD) Faculty of Medicine Alexandria University from September 2021 till June 2022.

In this study, the incidence of radiation-induced hypothyroidism was found to be 8.3% in breast cancer patients after 6 months of receiving loco-regional radiotherapy. This complication of hypothyroidism coincides with what is found in literature and it is one of the late side effects after radiotherapy to the neck, including the entire, or part of the thyroid gland. For example, Glatstein et al. revealed 11% incidence of hypothyroidism in lymphoma patients after receiving radiation [30]. Recent cohort study done in 2023 included 304 patients and also found somewhat similar incidence of 12% for RTIH [31].

Alterio et al. had much higher incidence for hypothyroidism in their study that included 73 patients, with 26% of them developing hypothyroidism. They also showed an increased incidence of hypothyroidism with female patients, which raises more concerns for breast cancer patients, as female patients represent the majority of breast cancer patients [19].

Two other study that dealt with hypothyroidism after mantle irradiation in Hodgkin’s disease (Fuks et al.,1976; Schimpff et al., 1980) revealed that thyroid affection slowly develops, and that up to 15% of patients developed dysfunction and a maximum of 66% was reached at around 6 years [32,33]. The incidence of hypothyroidism observed at 6 months may underestimate the true long-term incidence of radiation-induced hypothyroidism.

Another study by Laway et al., showed that TSH was significantly elevated after 3 months in those who had received neck irradiation [16]. Glatstein et al. also observed in their study elevated TSH within 12 months [30].

A recent Chinese study suggests the peak incidence of RIHT to be at 6–12 months, and a Dmean >21 Gy was the predictor for hypothyroidism [34].

As shown by Tamura et al. in their study on 126 lymphoma patients, there was an increased incidence of TSH elevation, from 26% after 2 years compared to 62% after 6–12 years of neck radiotherapy [35]. Two types of radiation induced damage were described; they are: sub-acute damage and late damage. Those two types may have different mechanisms associated with them. Hypothyroidism is associated with injury to tiny thyroid vessels and arteriosclerosis of the larger vessels, and further contribute to parenchymal damage of the thyroid cells, as well as, secondary capsular fibrosis [7].

In this study, the entire thyroid gland as well as the ipsilateral thyroid gland lobe were contoured, as the ipsilateral lobe usually receives most of the dose received by the gland. A thorough dosimetric evaluation was then made, which enabled us to correlate multiple factors with TSH levels of our patients.

Multiple factors that could affect the incidence of hypothyroidism were identified, such as maximum radiation dose, V40 to the thyroid gland and the ipsilateral thyroid lobe, as well as high mean dose and smaller volume of the ipsilateral thyroid lobe.

There is lack of consensus regarding the thyroid tolerance dose when it comes to radiotherapy, and most of the studies looked at hypothyroidism after radiotherapy in head and neck cancers. These studies suggested different causes linked to hypothyroidism, such as mean dose of 45 Gy, V30 of the thyroid gland, and smaller size of the thyroid gland [17,28,36,37].

When the data from our 109 patients was analyzed, there were multiple parameters that showed statistical significance. Patient factors included, ECOG PS, and smaller ipsilateral thyroid lobe. While dosimetric factors were thyroid maximum dose, thyroid V40 dose, ipsilateral thyroid lobe mean and maximum doses, as well as ipsilateral thyroid lobe V10, V15, V20, V25, and V40 doses.

Multivariate analysis showed that the most impactful factors were the percentage volume of the thyroid and ipsilateral thyroid lobe receiving 40 Gy, as well as the ipsilateral thyroid lobe volume in cc receiving 40 Gy (p values of 0.046, 0.044 and 0.031 respectively).

Receiver operating characteristic (ROC) curves were then used to identify cut-off points for these values, showing significance when thyroid V40 was higher than 5% with p value of 0.03, and ipsilateral thyroid lobe V40 more than 11% with p value of 0.036. As well as when more than 1 cc of the ipsilateral thyroid lobe received 40 Gy (p value = 0.031).

With the high prevalence of breast cancer among the female population, in addition to the fact that the survival is really high among these patients, it is of the utmost importance to determine all the treatment related side effects, including the late long-term side effects that may occur in these patients.

An important question is whether there is a relation between the radiation dose received by the thyroid and the thyroid dysfunction seen later in these patients. In addition to the even later side-effects that may include secondary malignancies of the thyroid, but this needs to be performed in future studies with much longer follow-up periods.

This study represents one of very few studies that were carried out to correlate between the radiation dose received by the thyroid gland and the affection of thyroid function after breast cancer radiotherapy. It is important to identify these patients in order to minimize the possible side-effects that could be caused by such dysfunction, and can be prevented with proper management.

It is also important to avoid this side-effect by properly determining dose constraints to the thyroid gland and being familiar with how to avoid unnecessary high doses that might affect it, using proper planning techniques.

Recent studies have had similar results to our findings, emphasizing the dosimetric threshold for thyroid injury. For instance, a study by Farshchian et al. (2022) reported that Dmax and V40 are critical dosimetric parameters for predicting hypothyroidism in breast cancer patients undergoing radiotherapy. Their findings showed that patients with higher Dmax and V40 had a significantly increased risk of developing hypothyroidism, similar to our results [38].

Additionally, research by Zhao et al. (2023) found that the incidence of hypothyroidism in breast cancer patients treated with hypofractionated radiation therapy was comparable to our findings. Their study highlighted the importance of routine thyroid function monitoring post-radiotherapy, suggesting a need for standardized screening protocols [34].

Further supporting our results, a meta analysis (2023) discussed the increased risk of hypothyroidism in breast cancer survivors, emphasizing the necessity of regular thyroid function tests. They also noted that the thyroid gland's exposure during radiotherapy could lead to long-term hypothyroidism, reinforcing the need for vigilant monitoring and preventive strategies [39].

In this study, possible risk factors were found that included high maximum radiation dose and V40 to the thyroid gland and the ipsilateral thyroid lobe, as well as high mean dose and smaller volume of the ipsilateral thyroid lobe, suggesting that contouring the ipsilateral thyroid lobe could be considered as it could be used as another dose constraint that helps with radiotherapy planning.

The likelihood of hypothyroidism was significantly increased with V40 of >5% of the thyroid gland, and V40 of >11% of the ipsilateral thyroid lobe.

Although DVH parameters are routinely assessed, specific thyroid dose constraints are not well established in breast radiotherapy. The identified thresholds may provide practical guidance for minimizing thyroid exposure in patients receiving supraclavicular irradiation.

### Limitations

Even though the current study provided clinically meaningful information regarding early thyroid dysfunction and its dosimetric predictors following hypofractionated locoregional breast radiotherapy, some limitations should be acknowledged. The follow-up duration was limited to 6 months and therefore may underestimate the true long-term incidence of radiation-induced hypothyroidism, which is known to develop over longer periods in some patients. In addition, the relatively small number of hypothyroidism events may limit the reliability of multivariate analysis. Larger studies with longer follow-up are warranted to further validate these findings.

## Conclusion

This study identifies Thyroid V40 > 5%, ipsilateral lobe V40 > 11% and V40 > 1 cc as practical thresholds for predicting early RIHT, suggesting routine monitoring of thyroid function after breast locoregional RT and offering potential constraints for 3D-CRT hypofractionated locoregional breast radiotherapy. Longer follow-up and larger studies are recommended.

## Supporting information

S1 Data Anonymized patient-level dataset used for the statistical analyses. (XLSX)
